# Supplementary material for: Feedback contribution to surface motion perception in the human early visual cortex
Source: eLife. 2020 Jun 4;9:e50933. doi: 10.7554/eLife.50933 (PMC7314553; doi:10.7554/eLife.50933)
Supplement: Transparent reporting form [file elife-50933-transrepform.pdf]

eLIFE
1st Floor
24 Hills Road
Cambridge CB2 1JP, UK
P 01223 855340
W elifesciences.org
T @elife

eLife's transparent reporting form

We encourage authors to provide detailed information *within their submission* to facilitate the interpretation and replication of experiments. Authors can upload supporting documentation to indicate the use of appropriate reporting guidelines for health-related research (see EQUATOR Network), life science research (see the BioSharing Information Resource), or the ARRIVE guidelines for reporting work involving animal research. Where applicable, authors should refer to any relevant reporting standards documents in this form.

If you have any questions, please consult our Journal Policies and/or contact us: editorial@elifesciences.org.

Sample-size estimation

- You should state whether an appropriate sample size was computed when the study was being designed
- You should state the statistical method of sample size computation and any required assumptions
- If no explicit power analysis was used, you should describe how you decided what sample (replicate) size (number) to use

Please outline where this information can be found within the submission (e.g., sections or figure legends), or explain why this information doesn't apply to your submission:

We based our sample size on previous studies, in particular the following ultra-high field fMRI studies on feedback effects across cortical layers available at the time, with sample sizes (in terms of number of subjects) between 4 and 12: Kok et al., 2016, *Current Biology*, 26(3), 371–376; Muckli et al., 2015, *Current Biology*, 25(20), 2690–2695; Klein, 2018, *NeuroImage*, 176, 301–312. The sample size is clearly stated in the *Methods* section, subsection *Experimental design* (p. 6).

Regarding sample size, it is worth noting that in the context of high-resolution fMRI research on low-level perceptual phenomena in early sensory cortex, measurement error (as opposed to sampling error) may be considered particularly relevant. It is reasonable to assume that low-level perceptual processes are well preserved across individuals and even across species. Thus, and because high-resolution fMRI data is noisy, the number of measurements has to be considered in addition to the number of subjects. (See Kolossa & Kopp, 2018, *NeuroImage*, 172, 775–785. https://doi.org/10.1016/j.neuroimage.2018.01.005).

Replicates

- You should report how often each experiment was performed
- You should include a definition of biological versus technical replication
- The data obtained should be provided and sufficient information should be provided to indicate the number of independent biological and/or technical replicates
- If you encountered any outliers, you should describe how these were handled
- Criteria for exclusion/inclusion of data should be clearly stated
- High-throughput sequence data should be uploaded before submission, with a private link for reviewers provided (these are available from both GEO and ArrayExpress)

eLife Sciences Publications, Ltd is a limited liability non-profit non-stock corporation incorporated in the State of Delaware, USA, with company number 5030732, and is registered in the UK with company number FC030576 and branch number BR015634 at the address 1st Floor, 24 Hills Road, Cambridge CB2 1JP | August 2014

Please outline where this information can be found within the submission (e.g., sections or figure legends), or explain why this information doesn't apply to your submission:

The number of participants and the number of measurements per participants is stated in the *Methods* section, subsection *Experimental design* (for the main experiment, see p. 6 & 8; for the control experiment, see p. 9-10). Criteria for data exclusion are described in the *Methods* section, subsection *Data acquisition & preprocessing* (p. 11). As the selection of a region of interest (ROI) based on anatomical and functional criteria is of critical importance in an fMRI study, a detailed description of the ROI selection can be found in a separate subsection (*Methods* → *ROI selection*). The functional and anatomical MRI data, experimental stimuli, and analysis code are all publicly available (see below, section *Additional data files*).

Statistical reporting

- Statistical analysis methods should be described and justified
- Raw data should be presented in figures whenever informative to do so (typically when N per group is less than 10)
- For each experiment, you should identify the statistical tests used, exact values of N, definitions of center, methods of multiple test correction, and dispersion and precision measures (e.g., mean, median, SD, SEM, confidence intervals; and, for the major substantive results, a measure of effect size (e.g., Pearson's r, Cohen's d)
- Report exact p-values wherever possible alongside the summary statistics and 95% confidence intervals. These should be reported for all key questions and not only when the p-value is less than 0.05.

Please outline where this information can be found within the submission (e.g., sections or figure legends), or explain why this information doesn't apply to your submission:

The statistical analysis is described in *Methods*, subsection *Hypothesis testing* (p. 16). All effects are reported in units of percent signal change (see *Methods*, *Data acquisition & preprocessing*, p. 11).

(For large datasets, or papers with a very large number of statistical tests, you may upload a single table file with tests, Ns, etc., with reference to sections in the manuscript.)

Group allocation

- Indicate how samples were allocated into experimental groups (in the case of clinical studies, please specify allocation to treatment method); if randomization was used, please also state if restricted randomization was applied
- Indicate if masking was used during group allocation, data collection and/or data analysis

Please outline where this information can be found within the submission (e.g., sections or figure legends), or explain why this information doesn't apply to your submission:

Our study employed a within-subjects experimental design.

Additional data files ("source data")

- We encourage you to upload relevant additional data files, such as numerical data that are represented as a graph in a figure, or as a summary table
- Where provided, these should be in the most useful format, and they can be uploaded as "Source data" files linked to a main figure or table
- Include model definition files including the full list of parameters used
- Include code used for data analysis (e.g., R, MatLab)
- Avoid stating that data files are "available upon request"

Please indicate the figures or tables for which source data files have been provided:

The functional and anatomical MRI data, experimental stimuli, and analysis code are publicly available. The fMRI dataset is available on Zenodo (https://doi.org/10.5281/zenodo.3366301). The software used for the presentation of retinotopic mapping stimuli, and for the corresponding analysis, is available on github (https://github.com/ingo-m/pyprf). Example videos of the main experimental stimuli are available on Zenodo (https://doi.org/10.5281/zenodo.2583017). If you would like to reproduce the experimental stimuli, the respective PsychoPy code can be found on github (https://github.com/ingo-m/PacMan/tree/master/stimuli/experiment). The respective repository also contains the analysis code and a brief description how to reproduce the analysis (https://github.com/ingo-m/PacMan). High-level visualisations (e.g. cortical depth profiles & signal timecourses) and group-level statistical tests are implemented in a separate repository (https://github.com/ingo-m/py_depthsampling/tree/PacMan).
